# Communication Strategies to Improve Child Participation in Triadic Consultations Within Healthcare Settings: A Scoping Review

**DOI:** 10.1111/hex.70853

**Published:** 2026-08-26

**Authors:** Maha Alharbi, Suzanne Grant, George Cherukara, Siyang Yuan

**Affiliations:** ^1^ School of Dentistry The University of Dundee Dundee Scotland UK; ^2^ Prince Sultan Military College for Health Sciences Dammam Saudi Arabia; ^3^ School of Medicine The University of Dundee Dundee UK

**Keywords:** child participation, communication, communication style, healthcare professional, paediatric, triadic consultation

## Abstract

**Introduction:**

Children under 18 are recognised as key stakeholders in healthcare and possess the right to actively participate in decisions regarding their care. Despite this, they are frequently relegated to passive roles during triadic healthcare consultations involving healthcare providers (HCPs), caregivers, and children. Enhancing child participation requires effective communication strategies; however, numerous barriers persist, including healthcare provider dominance and parental interference. This review aims to explore and identify effective communication strategies employed by healthcare providers and caregivers to improve child participation in triadic consultations within healthcare settings.

**Method:**

A scoping review methodology, based on the Joanna Briggs Institute framework and PRISMA guidelines, was used. A comprehensive search was conducted across four databases—PubMed, PsycINFO, Scopus, and CINAHL using predefined eligibility criteria. Studies focused on children under 18 in triadic healthcare consultations. Qualitative, quantitative, and mixed‐methods studies were included to map existing evidence and highlight gaps.

**Results:**

A total of 37 out of 41 studies revealed that child participation in triadic consultations is frequently minimal and varies significantly depending on the communication styles of HCPs and caregivers. HCPs exhibit a range of communication styles, with some adopting a directive or task‐focused (Dyadic approach) that prioritises medical tasks and often sidelines children in conversations, while others utilise supportive and facilitative styles (Triadic approach) that emphasise rapport‐building, open‐ended questioning, child‐friendly language, and the use of interactive techniques that facilitate engagement. Parental roles in this dynamic were classified into three main styles: supportive facilitator, dominant and protective. Factors such as the child's age and developmental stage, wider cultural influences, the emotional context, and the healthcare environment were also found to significantly impact child participation. A variety of measurement tools were utilised to assess participation levels, including the Roter Interaction Analysis System (RIAS), conversational analysis and context‐specific coding schemes. However, the review identified a notable lack of standardised tools, which limits the comparability of findings across different studies.

**Conclusion:**

This review highlights the need to enhance child participation in healthcare consultations through effective communication. However, improving participation requires more than communication skills training alone. HCPs must work closely alongside parents and caregivers to support children's participation rights and embed these principles in routine practice. Achieving this objective requires engaging both HCPs and parents to drive changes in clinical practice through a fundamental cultural shift in paediatric care delivery. Future research should employ validated measurement tools to evaluate progress and identify effective approaches to enhancing child engagement in healthcare settings.

**Patient and Public Contribution:**

The findings have been discussed with Patient and Public Involvement (PPI) members who have children. Their valuable insights contributed to the interpretation of the review findings and informed the next stage of the project, which will include audio observation of triadic consultations in paediatric dental settings.

## 1 Introduction

Children are recognised as essential stakeholders in their healthcare journey, possessing the right to participate actively in decisions regarding their care [1]. This right is affirmed in Article 12 of the United Nations Convention on the Rights of the Child (UNCRC), which states that children capable of forming their own views are entitled to express those views freely in matters affecting them and to have those views given due weight according to their age and maturity. The UNCRC further clarifies that this right explicitly extends to decisions about healthcare treatment and procedures [2, 3]. However, evidence from empirical research demonstrates that children are often afforded limited opportunities to participate meaningfully during healthcare consultations [4, 5]. The triadic nature of paediatric consultations, which involves healthcare professionals, caregivers, and children, presents unique opportunities and challenges for fostering meaningful child participation [6]. Effective communication to improve child participation in consultations is essential for fostering autonomy, reducing anxiety, and improving health outcomes [4]. Moreover, involving children in healthcare decisions fosters a patient‐centred approach, which helps tailor care to the specific needs of young patients and therefore improves their care experience [7]. This has been reflected in paediatric oncology, where evidence suggests that when children are included in discussions about their treatment plans, they are more likely to adhere to their treatment regimens and report better treatment satisfaction [8, 9]. Dental settings represent a particularly salient, if underexplored, example; paediatric dental care routinely involves invasive procedures performed on children, making meaningful participation especially essential.

Despite the recognised benefits of child participation, children's contributions to healthcare consultations are often limited [10, 11]. Much of the research has shown that both HCPs and caregivers frequently dominate health‐related conversations, leading to minimal verbal contributions from children [10, 12, 13]. Instead of initiating dialogue or expressing preferences independently, children primarily respond briefly to adult inquiries [5, 8]. This dynamic undermines children's autonomy and limits their understanding of health‐related information, potentially impacting long‐term health outcomes [7, 8]. Child‐related factors such as age, developmental stage, and individual characteristics are fundamental; younger children may require additional support and encouragement to express their thoughts and concerns, while older children may assert themselves more actively [8, 14]. Healthcare providers often adopt a paternalistic approach, which may inadvertently dismiss children's contributions and hinder the creation of an environment conducive to open dialogue [5, 15]. Caregivers may struggle to balance their roles as advocates while allowing children to voice their opinions. Achieving this balance is challenging, as healthcare providers or caregivers may dominate conversations or overshadow the child's voice while attempting to advocate for their best interests [16, 17]. Additional factors, such as cultural influences and the context of the healthcare consultation, further complicate these dynamics, underscoring the need for a comprehensive exploration of the multifaceted barriers and enablers to child participation in triadic consultations. For example, children are more likely to experience anxiety about dental appointments, which often involve invasive procedures, compared to other medical primary care consultations. This anxiety‐provoking environment presents specific challenges for dental professionals seeking to engage child patients. Therefore, this study aimed to explore the communication strategies that healthcare providers and caregivers use to improve child participation in triadic consultations within healthcare settings. The specific research questions are:

1. What does child participation look like during triadic consultations in healthcare settings?
2. What measures have been used to assess child participation?
3. What communication strategies and styles are used by HCPs and caregivers during triadic consultations?
4. Are there any other factors that influence children's participation in triadic consultations?

## 2 Methods

This review followed the JBI guidelines and was reported using the PRISMA‐ScR (Preferred Reporting Items for Systematic Reviews and Meta‐Analyses Extension for Scoping Reviews) framework [18, 19]. The review protocol was developed and internally reviewed before conducting this study [19, 20].

### 2.1 Eligibility Criteria

The eligibility criteria were guided by the research questions and the JBI approach using Population, Concept and Context (PCC) [21]. Inclusion and exclusion criteria were developed accordingly (Table 1).

- Population: child patients, caregivers and healthcare professionals
- Concept: child participation in triadic consultations
- Context: healthcare settings

**Table 1:** Inclusion/exclusion.

Criterion	Inclusion	Exclusion
Language	English	Non‐English
Type of study	Qualitative studies	Opinion/position papers
	Quantitative studies	
	Mixed‐Methods studies	
Population	Children under 18 years old with no learning difficulty and/or other behavioural problems	Children with learning difficulties and/or other behavioural problems
Concept	Child participation in triadic consultations	Caregiver or healthcare professional's participation in triadic consultations
		Dyadic or non‐triadic interaction
Context	Healthcare settings	Non‐healthcare‐related settings

#### 2.1.1 Type of Sources

This scoping review considered quantitative, qualitative and mixed‐methods studies of any design or approach for inclusion. Articles based on expert opinion (e.g., concerning what could be used or measured) were not included.

### 2.2 Search Strategy

Published papers were searched from 20/03/2024 to 29/07/2024 across four electronic databases: PubMed, PsycINFO, Scopus, and CINAHL, with no restrictions on the start date. The search strategy employed a combination of subject Headings (e.g., MeSH terms) and free‐text terms to capture the key concepts of (a) child and adolescent patients, (b) patient engagement and decision‐making, and (c) communication and consultation in healthcare settings. Boolean operators (AND, OR) were used to structure the search strings, and database‐specific subject headings were applied where appropriate to enhance precision and relevance. The development and execution of the search strategy were supported by an information specialist. A detailed list of search terms and strategies is provided in File S1.

### 2.3 Study Selection

All eligible papers were uploaded to EndNote 20. After removing duplicates, titles and abstracts were screened on Rayyan [22]. The full texts were screened by reviewers (MA, SY, GC, SG) independently against the selection criteria. Any disagreements that arose during the selection process were resolved through discussion.

### 2.4 Data Charting and Synthesis

A data extraction form was adapted from the Joanna Briggs Institute (JBI) to record key information relevant to the review questions. This included details on author name, year of publication, country, study design, healthcare setting, measurement of child participation, child participation description, adults' communication strategies/styles and factors influencing child participation. The extracted data were analysed using thematic analysis to identify common themes and patterns related to communication strategies and child participation.

## 3 Results

**Figure 1:**
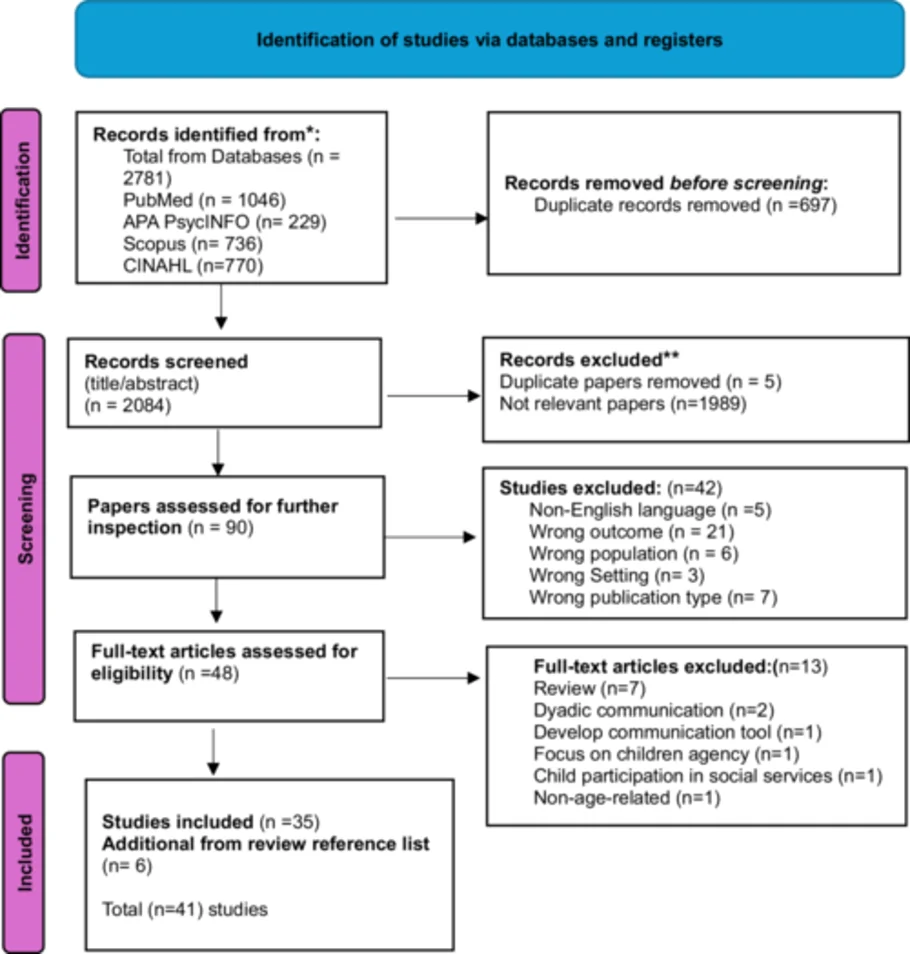
PRISMA‐SCR flow diagram of the study selection process. * Databases: PubMed, APA PsycINFO, Scopus, CINAHL. ** Exclusion made after title/abstract screening.

The search initially identified 2781 records and was reduced to 2084 after removing duplicates. 2084 records were screened by title and abstract, excluding 1989 irrelevant records and 5 duplicates. After screening by title and abstract, 48 articles remained for full‐text reading and screening. Thirteen studies were excluded, with 6 additional studies included, resulting in 41 studies in total (Figure 1).

### 3.1 Study Characteristics

In Table 2, the 41 included studies range from 1988 to 2024, with a significant increase in research conducted after 2005 (*n* = 29). Most of the articles were published in Western countries, including the United Kingdom.

**Table 2:** Overview of studies investigating child participation in triadic consultation using measurement.

Author (year), country	Aim	Study design	Healthcare setting	Type of measurement
Becker et al. (2018) [23], USA	To better understand relationships between observed communication and child‐reported perceptions of communication in a clinical setting.	Quantitative	Paediatric Gastroenterology Clinic	Roter Interaction Analysis System (RIAS)
Savage and Callery (2007) [8], Ireland	To explore the perspectives of children and parents on communication with healthcare professionals regarding dietary management of cystic fibrosis	Mixed‐Methods	Nursing practice	Discourse Analysis (Communication Patterns)
Coyne et al. (2016) [4], Ireland	To examine participants’ views on children's participation in information‐sharing and communication interactions in paediatric cancer care.	Qualitative	Paediatric Cancer Unit	NA
Chaudhuri et al. [24], India	To understand professionals’ views regarding information exchange during the cancer treatment of children, complement these findings with clinic‐based ethnographic observation of real‐life consultations.	Qualitative	Paediatric oncology	NA
Bray et al. (2023) [25], UK	To identify the key concerns and information needs of young people with AIS and their parents when attending spinal clinics, and examine what resources or interventions might help improve young people's ‘participativeness’ and health literacy during clinic consultations	Qualitative	Spinal clinic	NA
Forner et al. (2020) [26], USA	To investigate SDM‐related outcomes through turn analysis and an assessment of patient‐centred dialogue.	Mixed‐Methods	Paediatric otolaryngology	Roter Interaction Analysis System (RIAS) (Utterance)/Turn Analysis (Turn Taking and Sequence/Patient Centeredness Score/SDM Questionnaire
Bhatti et al. (2021) [27], UK	To explore the familiar facilitators of delivering oral health advice from dental teams, parents’ and children's experiences, to identify and inform practical recommendations for clinical practice.	Qualitative	Dental practice	NA
Baggens (2001) [28], Sweden	To explore the content of the conversations and analyse its relationship to both the child health promotion programme and the question of which party decides what is talked about in these encounters	Qualitative	Child health centre	Content‐Oriented (Utterances)
Ekberg et al. (2022) [29], Australia	To explore how clinicians and parents/guardians direct their conversations with a child patient when they are present in a consultation.	Qualitative	Paediatric palliative care	Conversation Analysis
Aronsson and Rundström (1988) [30], Sweden	To examine how parents regulate their children's discourse during paediatric consultations	Qualitative	Allergy outpatient clinic	Child Allocated Turns (CATS)/Conversation Analysis (Discourse Space, Turn‐Taking, Content of Speech)
Cox et al. (2007) [31], USA	To examine how a child's, physician's, and parent's gender and visit length affected participation in paediatric visits.	Quantitative	Paediatric clinic	Roter Interaction Analysis System (RIAS) (Utterances)
Coyne (2006) [5], UK	To explore the views of children, parents, and nurses on children's participation in their care within a healthcare setting.	Qualitative	Paediatric hospital setting	NA
Bari et al. (2017) [32], Pakistan	To analyse the communication skills of paediatric postgraduate residents during clinical encounters using video recordings.	Qualitative	Children's Hospital (hospital auditorium)	Paediatric Consultation Assessment Tool (PCAT) And Arizona Clinical Medical Interview Rating Scale (ACIR)
Barber et al. (2019) [33], UK	To determine if the current care pathway for hypodontia promotes shared decision‐making.	Qualitative	Orthodontic clinic	NA
Cahill and Papageorgiou (2007) [34], UK	To identify what factors in the interaction between a doctor, a child (aged 6–12), and their carer during a primary care consultation are associated with the child's participation.	Qualitative	Paediatric Primary Care	Conversation Analysis/Non‐Verbal Communication
Hui et al. (2022) [6], China	To understand how children's understanding of information is elicited during paediatric genetic counselling sessions.	Qualitative	Paediatric genetic clinic	Discourse analysis approach.
Inman (1991) [35], UK	To explore three main areas: Children's perspectives on the clinic environment.	Mixed‐Methods	Oncology clinic	NA
	Parents’ opinions on how impending clinic appointments affect their child's behaviour.			
	Observations of the interactions during clinic consultations.			
Jenkins et al. (2020) [36], UK	To examine how children instigate talk during their medical consultations and whether their contributions are successful.	Qualitative	Paediatric allergy clinic	Conversation Analysis
McPherson and Redsell (2009) [37], UK	To explore how healthcare practitioners in UK primary care involve children in asthma consultations.	Mixed Method	Paediatric Primary Care	NA
Meeuwesen and Kaptein (1996) [38], Netherlands	To investigate changes in the conversational patterns during medical interviews between doctors, parents, and children over time, focusing on the child's conversational contribution and the organisation of the conversation.	Qualitative	General practitioner's surgery	Turn Allocation System (TAS)
Rawdon et al. (2020) [39], UK	To identify how adolescents living with Type 1 diabetes and their parents communicate about Type 1 diabetes management with healthcare professionals in a clinical setting.	Qualitative	Outpatient clinics	NA
Sleath et al. (2011) [40], USA	To investigate how often caregivers and children asked questions about asthma management during paediatric appointments.	Quantitive	Paediatric primary care practice	NA
Stivers (2012) [41], USA	To examine the factors that predict whether a child will answer a physician's question during a primary care visit.	Mixed Method	Community practice	Measure Child Verbal Responses
Tates et al. (2002a) [15], Netherlands	To explore the relationship within this triad by developing a typology of doctor‐parent–child interactions.	Quantitive	General Practitioner's surgery	Three‐Point Scale (1: Active Involvement, 2: Passive Involvements, 3: No Display of Involvement)
Tates et al. (2002b) [16], Netherlands	To explore the nature of communication in the doctor‐parent‐child triad at the general practitioner's surgery.	Qualitative	General Practitioner's surgery	Roter Interaction Analysis System (RIAS)
Tates et al. (2002c) [17], Netherlands	To describe how the three participants display their orientation to their institutional roles and identities, how they collaboratively co‐construct the course of action, and how these discursive	Qualitative	General Practitioner's surgery	Conversation Analysis
	Constructions structure the ongoing interaction.			
Tates and Meeuwesen (2000) [10], Netherlands	To explore turn‐taking patterns in doctor‐parent–child communication to understand asymmetry and control dynamics.	Quantitive	General Practitioner's surgery	Turn Allocation System (TAS)
Taylor et al. (2010), UK	To investigate child and carers’ attitudes towards child involvement in paediatric consultations.	Qualitative	Inpatient and outpatient departments.	NA
Tran et al. (2023) [16], USA	To explore the dynamics of triadic paediatric healthcare communication to understand how healthcare providers and parents can facilitate or hinder children's involvement in their own asthma and allergy care.	Mixed‐Methods	Paediatric clinic	Conversation Analysis
Vigilante et al. [12], USA	To explore the quantity and types of communication by children during their subspecialty healthcare visits.	Quantitative	Paediatric outpatient clinic	RIAS Coding Scheme. (Roter Interaction Analysis System)
Wassmer et al. (2015) [42], UK	To describe and evaluate the nature of communication between doctors, parents, and children during paediatric outpatient clinic visits.	Quantitative	Outpatient paediatric clinic	Modified Version of Roter's Process Analysis System.
Williamson et al. (2004) [43], USA	To examine questioning practices in racially discordant interactions and describe how these practices engendered child‐centred care	Qualitative	Paediatric oncology	Conversations Analysis
Yuan et al. (2019a) [44], UK	To describe the development and refinement of a coding scheme to record the triadic communication between dental professionals, child patients and parents	Quantitative	General dental practice	Paediatric Dental Triadic Interaction Coding Scheme (Paed‐Trics)
Yuan et al. (2009b) [14], UK	To investigate the interaction between child, parent, and dental nurse, and to determine the effect of nurse and parental behaviours on child participation in an oral health promotion session.	Quantitative	General dental practice	Paediatric Dental Triadic Interaction Coding Scheme (Paed‐Trics)
Callery and Milnes (2012) [45], UK	To examine communication between nurses, children, and parents during asthma review consultations.	Qualitative	Paediatric respiratory outpatients’ clinics, community children's nursing services and general practices	Conversation Analysis (Turn‐Taking‐Initiation‐Types of Utterances)
Cox et al. (2009) [11], USA	Evaluate how participation changes with the child's age and examine the effect of parental education on participation.	Quantitative	Physician offices and paediatrician offices	Roter Interaction Analysis System (RIAS)
Van Dulmen (1998) [46], Netherlands	To analyse verbal and non‐verbal communication between paediatricians, parents, and children during outpatient encounters.	Quantitative	Consecutive outpatient	Roter Interaction Analysis System (RIAS)
Wissow et al. (1998) [47], USA	To examine communication patterns during emergency care for childhood asthma and investigate if a ‘patient‐centred’ provider style was linked to higher parent satisfaction and increased parent and child participation.	Quantitative	Emergency departments	Roter Interaction Analysis System (RIAS)
Runeson et al. (2002) [48], Sweden	To describe the degree to which hospitalised children participate in care decisions and compare this with what was considered optimal participation.	Qualitative	Inpatient hospital setting	(Scale Of Participation in Decision‐Making)
Nova et al. (2005) [5], Italy	(1) To explore how and with what specificity young patients contribute to paediatric visits.	Qualitative	Outpatient paediatric clinic	Content Analysis/Discourse Analysis
	(2) To examine the communicative and relational ways adults handle children's interventions during these visits.			
Ruhe et al. (2016) [9], Switzerland	To explore the perspectives of children and adolescents with cancer on their participation in discussions and decision‐making related to their diagnosis and treatment.	Mixed Method	Paediatric oncology centres	NA

(*n* = 14) and the USA (*n* = 10). Additional studies came from the Netherlands (*n* = 6), Sweden (*n* = 3), Ireland (*n* = 2), and one each from Australia, Pakistan, Italy, China, Switzerland, and India.

54% of the included studies used a qualitative design (*n* = 22). 29% of studies were quantitative observational studies (*n* = 12), while 17% of studies used mixed methods designs (*n *= 7). In terms of settings, 37 out of 41 studies were conducted in medical settings, while 4 were conducted in dental settings. Participants' ages ranged from early childhood to adolescence, with 10 studies specifically targeting ages 4–12 years.

### 3.2 Key Findings

#### 3.2.1 Child Participation in Triadic Consultations

From Table 3, 37 out of the 41 studies indicate that child participation in triadic consultations is frequently limited, with many children exhibiting low levels of verbal engagement [4, 5, 6, 8, 10, 11, 12, 13, 15, 16, 17, 23, 24, 26, 27, 28, 29, 30, 31, 32, 33, 34, 35, 36, 38, 39, 40, 41, 42, 43, 44, 45, 46, 47, 48, 49, 50, 51]. Child participation varies significantly based on child characteristics such as age and developmental stage [4, 6, 48, 49]. Younger children tend to be more passive, relying on caregivers for communication, while older children and adolescents actively engage in discussions about their health [8, 13, 24, 44]. Younger children aged 2–6 years often exhibit minimal verbal engagement during healthcare consultations [48, 49]. Their contributions are typically limited to brief responses when prompted or when expressing personal feelings [13, 44, 48, 49, 50]. They may engage in non‐verbal communication, such as nodding or looking away, indicating discomfort or disinterest [6, 36]. Their ability to articulate symptoms or ask questions is often restricted due to their developmental stage [28]. Studies have shown that children only spoke about 6.7% of the time during subspecialty visits [12, 16]. Additionally, in some cases, children's verbal contributions comprised as little as 4%–6.7% of the total conversation during consultations [11, 47]. Most interactions are characterised by minimal responses or non‐verbal cues. For instance, a 4‐year‐old may respond to a healthcare provider's question with a simple ‘yes’ or ‘no’ and may not initiate further dialogue [4, 13, 48, 49].

**Table 3:** Adults' communication strategies and factors influencing child participation.

Author (year)	Child participation	Adults’ communication strategies	Influencing factors of child participation
Becker et al. (2018) [23]	Low child participation with limited verbal participation	Physician facilitative communication (e.g., asking questions, physician partnership) and affective communication (e.g., chit‐chat, rapport, jokes). Parents’ affective communication (e.g., chit‐chat) and instances where parents interfered with child participation by answering questions directed at the child	Child's age, social conversation, visit characteristics (less acute or complex), physician and parent interference (perceived lack of interest, time restrictions). Physician facilitative strategies
Savage and Callery (2007) [8]	Children limited the extent to which they could express their views, resulting in brief and closed answers to questions asked (children provided brief and closed answers)	The physician's dominant approach was primarily directed towards the parents. The surveillance approach involved an interrogative style of questioning. Parental dominance (interrupted their children's responses, leading professionals to redirect conversations to parents; parents’ accounts were privileged over children's accounts	Child's age, parents’ styles (interrupt, privileged over children's account), professional Communication Style (an interrogative style)
Coyne et al. (2016) [4]	Younger children were described as passive participants; children aged 11 and above were more likely to ask questions, provide information about their symptoms, and actively participate in discussions	Oncologists maintain an open and honest approach, a hopeful and spiritual approach, and manage restricted information sharing. Parents’ approaches varied from supporting full disclosure to filtering information to protect their children from distress	Child's age, parents’ Communication Style, and Healthcare Provider's Strategy. cultural factors (Children trusted and valued their parents’ role as interpreters of information, advocates, and communication buffers.)
Chaudhuri et al. (2022) [24]	Children under 5 had minimal active involvement, whereas older children and adolescents displayed greater participation by asking questions and using nonverbal cues during discussions	Oncologists and nurses used empathetic and open communication between clinicians, parents, and children with cancer. Developmentally appropriate communication methods (language based on the child's age and understanding) were used. Parents play a crucial role in filtering and explaining information to their children	Child's age and maturity, parental control and concerns, emotional environment Healthcare professional's communication approach
Bray et al. (2023) [25]	NA	Healthcare Providers addressed young people directly and provided clear, age‐appropriate information; medical jargon and complex explanations made it difficult for young people to comprehend the discussions fully. Parent Dominant Role (led conversations, asking most questions and controlling the flow of information) Supportive Behaviour (acted as a facilitator by clarifying and simplifying information)	Child's anxiety and uncertainty, Healthcare professional's experience and preparation (e.g., prompt sheets or educational resources), and Parental involvement.
Forner et al. (2020) [26]	Children's participation was minimal (the child spoke less per turn compared with a physician)	Surgeons dominated the consultation regarding talking, mostly in a unidirectional manner. (medical task‐focused). Parents had limited interaction and were involved primarily as respondents to the physicians’ questions, and were secondary in initiating dialogue	Physician dominance, Medical Setting Differences (Primary care consultations are typically shorter and focus less on lifestyle and general health compared to surgical specialities)
Bhatti et al. (2021) [27]	Children aged 7–10 years often had minimal direct involvement (they demonstrated interest and could speak during their interactions, such as when given direct advice)	Doctors’ directive Approach (often speaking to parents more than to children), Interactive Techniques: using visual aids and child‐focused communication. Parents as information Interpreters, supportive roles	Child's age, clinical supportive environment and Parental involvement
Baggens (2001) [28]	Children were often passive (children introduced very few topics into the conversation, bringing up topics related to their surroundings and using them as ‘tickets’ to engage in conversation)	Nurses dominated the interaction by initiating most conversation topics. The nurses invited the parents to ask questions. Parents act as intermediaries in the communication and might limit initiation	Child's age, nurse's role, consultation agenda, and visit routine
Ekberg et al. (2022) [29]	Tag questions invited participation from the child, whether verbally, vocally or body‐behaviourally, without making any absence of such involvement. accountable	Healthcare Providers use a tag question directed at children during the consultations to address the children's understanding of their experiences. Parents’ protective Involvement, who sometimes managed interactions to shield their children from stress or discomfort during discussions	Child's abilities to express their feelings and concerns (verbal and nonverbal), and the complexity of the condition
Aronsson and Rundström (1988) [30]	Children's participation was significantly limited (Children occupied only 8% of the total discourse space)	Doctors’ Directive and Task‐Focused: Doctors controlled the structure of the consultations, directing most of their communication towards parents rather than children: Efforts to Engage Children. Parents: High‐Control Parents (Frequent Turn Appropriation, speaking for the Child, Third‐Person References), Low‐Control Parents (Facilitate Child's Communication, act as ‘Cultural Brokers’, and support child's contributions)	Parental Control, doctor–parent dynamic, complexity of medical framing (Children's lack of familiarity with medical terminology and the context of consultations)
Cox et al. (2007) [31]	Limited active involvement (47% of children gave information,15% gathered information, 46% spoke relationship‐building talk)	Doctors’ facilitative communication, such as open‐ended questions, age‐appropriate language, building rapport or socioemotional behaviour. Parents who may intervene in conversations, impacting child participation	Child's age and gender, Visit length, Physician's gender
Coyne (2006) [5]	Passive s (often limited) child participation	Nurses may sometimes use an impersonal, business‐like approach, using confusing technical terms, ignoring the child, or discussing matters with other doctors or parents in front of them. Due to workload pressures, they may have limited time to explain information clearly. Parents act as facilitators and interpreters for the child	Child's age and cognitive maturity, child's previous experience, personality, severity of illness, clinician's workload, time constraints, hospital structures and routines (ward culture, limited child‐friendly resources, parental presence)
Bari et al. (2017) [32]	Limited involvement of children	Doctors’ Instrumental behaviour occurs when the doctor takes a leading role in the interaction, treating the patient as a medical object. This dynamic can reflect a power imbalance, where the doctor is in control, and the child's participation is ignored, often disregarding non‐verbal cues or attempts to engage. Parents or Guardians self‐appointed representative of the child	Physician's Dominant role, Marginal nonverbal communication, Documentation focus, and Parental mediation
Barber et al. (2019) [33]	Adolescents were often involved minimally, primarily contributing by outlining their symptoms or expressing concerns	Healthcare Providers introduction by team members, use of child‐friendly language, non‐dental examples and props to improve explanations, humour to alleviate anxiety and multiple opportunities for parents and young people to ask questions. Parents who play a supportive and advocacy role	Clinician approach, Time constraints, Parental role, organisational limitation
Cahill and Papageorgiou (2007) [34]	Limited Verbal Participation (children only contributed to about 5.42% of the total verbal utterances in a consultation.) Responding to Invitations, Slower Response Times	Healthcare Providers Direct invitations to speak, doctors directly invite children to speak, asking questions or prompting them to share their thoughts or feelings. Addressing the child directly: When doctors addressed the child by name and made eye contact, the adult carer was less likely to answer on the child's behalf. Triadic Talk: When the seating arrangement allowed for a triangular setup where the doctor, child, and carer were equally spaced, there was a greater tendency for three‐way conversations to occur. Parents often act as interpreters and advocates, provide emotional support and contribute to shared decision‐making	Seating arrangement, Doctor's communication style, Parent communication style
Hui et al. (2022) [6]	Limited active involvement: Children often provided minimal responses like brief verbal (e.g., Yeah) and nonverbal (nodding, shaking, smiling)	Healthcare Providers direct questioning, such as probing into children's epistemic access to relevant information in the past or an invitation to recall information. Parents Limited Facilitation (Parents often align with the assumptions of medical professionals regarding their child's understanding)	Child's age, maturity and capacity to understand, limited time that allowed child and parents for selecting treatment option, parental communication style
Inman (1991) [35]	Children's participation in oncology consultations was minimal.	The doctor's dominant role and limited direct engagement. Parents’ Protective and Mediating Role (Parents often acted as intermediaries, speaking on behalf of their children or interpreting questions)	Clinic environment, child's perception of treatment, attitudes of parents and children before attending a medical appointment, parental communication style and preparation
Jenkins et al. (2020) [36]	Children's non‐solicited Talk necessarily occurs when they are not currently active participants	Doctors’ Responsive (verbal) strategies (summons and prosodic variations) and non‐verbal resources (tapping and gaze) to break into the interaction. Parents Primary Recipients (Children most often directed their talk to parents rather than the doctor). Supportive role: validated the child's input and facilitated the child's contribution	Child's age and development, complexity of medical language, and parents’ communication style
McPherson and Redsell (2009) [37]	NA	NA	Child's Age and cognitive ability, visit duration, parental behaviour and time constraints, HCP Training and Confidence
Meeuwesen and Kaptein [38]	Children are substantially involved in Medical visit communication, but their participation level is not high	General Practitioner Directly Addressing the Child: GPs increasingly direct their questions and comments to the child rather than exclusively communicating through the parent. Giving more space to the child: doctors are affording more conversational space to the child, even if it means the parent speaks a little less. Parents Parental Interference, Supportive and Directive	Child's age and cognitive level, parental control, type of illness and nature of the location (e.g. GP's surgery or paediatric department)
Rawdon et al. (2020) [39]	Adolescents’ participation varied based on their roles in communication	Communication was categorised into three main patterns: parent‐led, collaborative, and adolescent‐led. Healthcare Professionals: Supportive and Direct Questions, Reassurance and Guidance. Parents’ Dominant Role in Parent‐Led Interactions, Facilitators in Collaborative Sessions	Child's age and developmental stage, Adolescent autonomy and self‐management skills, parents and healthcare professional interaction dynamics and clinical environment
Sleath et al. (2011) [40]	During longer visits, children were more likely to ask one or more questions about asthma medications	Healthcare Providers: Directive and Closed‐Ended Questions (Providers asking for and including child and caregiver input into the asthma management treatment plan). Limited Inclusion. Caregivers: Supportive but Directive approach	Visit length, Provider characteristics, and provider and caregiver dynamics
Stivers (2012) [41]	Children's participation was generally low. Children were more likely to respond to simpler, yes‐no questions or questions that invited minimal response	Physician's Directive Style with Selective Engagement (Physician Gaze, Question Type, Early Social Questions) Parents’ Intervention and Turn‐Taking (Parents often intervened, especially if the child delayed responding.) Mediation style	Child's Age, child gender, question content and format, physician gaze, multiple caregivers, addressing child terms
Tates et al. (2022a) [15]	Active involvement (when the child took the initiative or actively responded to the adults), passive involvement (e.g. by nodding when the parent answered the question or by seeking eye contact), no display of involvement (when the child did not respond to adult behaviour or display non‐verbal lack of involvement, e.g. by looking around when being addressed)	General Practitioners Supportive GP behaviour (directing medical questions at the child and involving the child in the discussion. Non‐supportive GP behaviour (directing most questions relating to medical conditions and discussing them with parents). Supportive parents, by supporting both verbally and nonverbally, encourage the child to take an active role in the medical interaction, remaining in the background and leaving the child enough room to respond to questions. non‐supportive parents (tend to speak to the child, ignoring the child's contributions or by interrupting doctor‐child interaction)	Child's Age, child behaviour, and parental supportive behaviour
Tates et al. (2022b) [16]	Limited and passive participation	General Practitioners’ Affective Behaviour:	Child's age and characteristics, time, parental mediation, and GP communication style
		1. Social behaviour and partnership building	
		2. Involvement (verbal attention, disagreement)	
		Instrumental behaviour:	
		3. Counselling and advice	
		4. Medical information exchange	
		5. Psychosocial information exchange	
		6. Consultation structuring and clarification	
	v	Parents Supportive but Dominant Role	
Tates et al. (2022c) [17]	low degree of child participation (verbal and non‐vervbal)	General Practitioners Doctor's Alignment with Parents:(Doctors may unintentionally reinforce the parent's dominant role by primarily directing questions and responses to them, even if initially aiming for more child involvement). Lack of Meta‐communication: (that doctors don't engage in ‘meta‐communication’, meaning they don't comment on or try to adjust the communication dynamics to encourage more child involvement). Parents’ role (Parents often assume the role of the spokesperson, speaking for the child rather than allowing them to speak directly to the doctor)	Child's age, parental mediation, GP's communication style, Institutionally Co‐constructed (limited participation is not solely due to the child's age or ability, but is shaped by the roles and expectations established within the doctor‐parent‐child dynamic)
Tates and Meeuwesen (2000) [10]	Children's participation in medical consultations is limited. (Children occupied only a small portion of the conversation space, often participating only when addressed directly or during the physical examination segment)	Doctors tend to adjust their communication style based on the child's age, giving older children more space and opportunities to participate. Parents’ dominant and mediating role, supporting their child's response by providing clarification	Child's Age and personality, health condition, previous experiences, parental control, and different stages of the consultation process, 3 segments (segment 1: medical history, segment 2: physical examination, segment 3: diagnosis and prescription)
Taylor et al. (2010) [53]	NA.	NA	Both parents and children identified three factors: age, maturity and communication
			Skills of the child as a factor. Children identified one other factor, ‘feeling at ease’, which they perceive as influencing their involvement in the consultation. Parents identified a further seven factors: gender, cognitive ability, confidence, family dynamics, doctor's judgement and Illness severity.
Tran et al. (2023) [16]	Child participation was minimal, representing only 13% of the interaction	Healthcare Providers directly address the child but often use medical jargon and interrupt both parents and children. Parents speak on behalf of their child, interrupting both the provider and the child	Using technical language, clarification, parental interruption, and formation of dyadic coalitions
Vigilante et al. (2015) [12]	Low child engagement. Children only spoke about 15% of the time during their subspecialty visits	Healthcare Providers Directive communication and Informative Approach, Limited Rapport‐Building. Parents’ Dominant Role in Conversations	Child's age, Child's race/ethnicity, cultural factors, type of encounter(topic), caregiver presence
Wassmer et al. (2004) [42]	Children's direct verbal contribution was minimal. They asked for less information (3%) and had more social conversation (19%). Doctors dominated turn‐taking (52%), while children took 9% of all turns	Physicians 84% instrumental communication (e.g. asking questions, giving information or instructions), 13% affective behaviour (expressing concerns and worries) and 3% social (small talk). Parents’ Dominant and Informative Role (giving information (83%), seeking information (13%) and Social (4%)	Child's Age. Consultation Type (follow‐up or a new patient visit), Doctor's Talk Time, Parent as a Mediator
Williamson et al. (2004) [43]	Acting as both recipients and respondents to questions from both physicians and caregivers.	Paediatric Oncologists Question‐Based Engagement. (used questioning practices to encourage children to participate in conversations) Directive and Cooperative Approaches (built upon each other's talk to enable the child's conversational turn and reformulated requests as questions to invite the child's permission and cooperation.) Parents supporting their child's responses	Type of Question Posed, Caregiver Dynamics, Situational Context
Yuan et al. (2019) [44]	Children's verbal behaviours were developed simply as speech ‘yes’, ‘speech no’, ‘speech another dental talk’, ‘laugh’, and’ crying. Nonverbal behaviour of children's distress behaviours (e.g. ‘withdraw’, ‘pushes away (hand)’, ‘turns head’)	Dental Professional's directive and Supportive Communication (Dental professionals frequently used ‘dentally engaging talk’ and ‘praise’ to involve children and encourage cooperation. Techniques like the ‘tell‐show‐do’ (TSD) were common, aiming to reduce anxiety by explaining and demonstrating procedures before performing them. Instruction and Praise: Providing clear instructions and positive reinforcement. Parents Facilitating Role (Parents often engaged in ‘verbal facilitation’, helping bridge the communication between the child and the dental professional by rephrasing or explaining questions in simpler terms). Supportive Communication: Parents sometimes praise or reassure children to create a positive environment and encourage their participation	Child's Emotional State, Child's Comprehension and Previous Experiences, Consultation Context, Parental Mediation
Yuan et al. (2019) [13]	Children often needed prompts to contribute actively, and their participation was mostly in the form of simple verbal responses such as ‘yes’, ‘no’, or brief explanations related to oral health habits	Dental professionals use child‐centred communication strategies such as ‘dentally engaging talk’, social talk, jokes, and praise to encourage participation. Parents Parental Facilitation: by rephrasing the nurse's questions or adding context that the child could understand. Praise and Joking: used praise and humour to engage their children and foster a supportive environment	Child's Age, Consultation Length, Parental Role, Consultation Dynamics
Callery and Milnes [45]	Children were prompted to participate through direct questioning, but their contributions were typically brief and dependent on the questions posed	Nurses employed both directive and supportive communication. They initiated consultations with open‐ended questions directed at the child to engage them in discussing their symptoms or treatment. Nurses used neutral responses and re‐directed questions. Parents answer questions directed at the child and raise concerns	Child's Age and Development, Asthma Knowledge and Experience, Power Dynamics
Cox et al. (2009) [11]	Limited Participation in Their Care, Child talk comprised 6.7% of the visit talk	Healthcare Providers: Adjusting Communication‐Based on Age, Directive Interaction, Parents’ Parental Interruptions	Physician communication style, Parent‐Child Talk Dynamics, Child age, Parental education
Van Dulmen (1998) [46]	Children's contributions to outpatient encounters were limited to 4%	Paediatrician: Task‐related approach (instrumental) rather than focused on relationship‐building (affective). Paediatricians were more likely to address social and psychosocial topics to children directly, but medical information was still predominantly directed at the parents. Directive Approach (asking medical questions and providing information primarily to parents). Parents’ dominant role: Parents often respond on behalf of their children	Child's age and gender, order of the medical visit (Initial vs. Follow‐Up Visits), type of disorder
Wissow et al. (1998) [47]	Children spoke little to a provider (an average of 20 statements per visit compared to 156 by parents). Children contributed an average of 43 utterances per visit	Healthcare Providers Patient‐Centred Style: When providers used a patient‐centred style (informative, asking about concerns, actively listening), parents and children participated more. Biomedical Focus: focused on medical aspects, with limited attention to psychosocial needs. Parents’ Gatekeeping Information/Speaking for the Child	Child's Age and Developmental Stage, Severity of Asthma Exacerbation, Anxiety and Stress
Runeson et al. (2002) [48]	Varied child participation: ranging from active involvement, passivity and weak questioning to Strong protests, but also by losing control over the situation and kicking and screaming in panic	Healthcare Providers informed children about what would happen without offering choices or actively seeking their input. Parents, the non‐supportive role of their children is to advocate for their preferences or participate in decisions	Child's Age and Developmental Stage, Complexity of the Decision, Child's Personality and Preferences, and Hospital Routines
Nova et al. (2005) [5]	Children's overall involvement was quantitatively low, comprising only about 10% of the total communication during visits. Children aged 2–6 engaged in verbal interactions when prompted or when they chose to express personal feelings or ask questions	Healthcare Providers and Parents: (Integrating vs. Non‐Integrating Responses) Integrating Responses: Adults acknowledged, validated, and elaborated on children's contributions, fostering further participation	Child's Age and Developmental Stage, Child's Personality, Familiarity with Healthcare Setting
		Non‐Integrating Responses: Adults dismissed, ignored, or spoke over children, potentially discouraging future participation attempts	
Ruhe et al. (2016) [9]	NA	NA	Child's Age and Developmental Stage, Child's Health Status, Complexity of the Decision, Child's Preferences, Information Provision, and Supportive Environment

Children aged 7–11 years begin to show increased verbal participation [8, 24], with studies showing that their verbal engagement accounts for 5.4‐8% of the total consultation [6, 12, 23, 30, 34]. They are more likely to ask questions and share information about their symptoms [8, 24]. They may engage in both verbal and non‐verbal cues, such as raising their hands to ask questions, nodding when their parents answer questions or using facial expressions to convey feelings about their health [29, 42, 43, 45]. Older children and adolescents are generally more inclined to participate actively in consultations [8, 24]. They often express their thoughts and preferences more clearly and assertively [8, 24]. They may engage in detailed discussions, provide comprehensive explanations of their symptoms, and express their opinions about treatment options [33, 40]. Non‐verbal behaviours may include maintaining eye contact and using gestures to emphasise points. Research indicates that adolescents can account for approximately 13‐15% of the total communication during consultations [12, 16, 49]. Adolescents are more inclined to express their thoughts, preferences, and concerns as well as actively participate in the treatment plan during consultations [4, 15, 39, 40].

#### 3.2.2 Measures to Assess Child Participation

Only 29 studies reported varying assessment measures to capture child participation in different healthcare settings. A limited number of measurement tools were used more often, with 8 studies using the Roter Interaction Analysis System (RIAS) [11, 12, 15, 23, 31, 42, 46, 47] and 14 studies applying different approaches of the discursive analysis approaches [6, 8, 10, 16, 17, 28, 29, 30, 34, 36, 38, 43, 45, 50]. Two studies used customised coding schemes for specific settings, such as Paediatric Dental Triadic Interaction Coding Schemes [13, 44]. The rest of the studies used different instruments to assess child participation during healthcare consultations, such as the Paediatric Consultation Assessment Tool (PCAT) and the Arizona Clinical Medical Interview Rating Scale (ACIR), as well as a three‐point scale, which were mainly used to measure either shared decision‐making or children's voices being heard [26, 32, 41, 48, 49].

1. **Roter Interaction Analysis System (RIAS)**
  The RIAS tool has been used across eight studies by coding communication behaviours and quantifying children's verbal contributions in consultations [11, 12, 15, 23, 31, 42, 46, 47]. RIAS is a useful tool for analysing communication dynamics in healthcare consultations [52]. It categorises the types of utterances made by the child, parents, and healthcare providers [15, 46]. For instance, if the child asks questions about their symptoms or expresses concerns about a treatment, these contributions are coded as ‘child‐initiated questions’ [31] This analysis can reveal how actively the child participates in the conversation, pinpointing areas where they feel confident to participate and those where they may be reluctant to contribute [47].
2. **Discursive Analysis Approaches (Conversation Analysis, Discourse Analysis and Content Analysis)**
  Conversation analysis was used in eight studies to assess the structure of triadic interactions, focusing on turn‐taking and the flow of dialogue [16, 17, 28, 29, 34, 36, 43, 45]. Studies by Jenkins et al and Ekberg et al employed this method to examine the nuances of how children, parents, and healthcare providers navigate communication during consultations [29, 36]. Conversation analysis enabled researchers to capture subtle dynamics, such as who initiates topics, the sequence of turns, and the influence of adults in guiding or limiting child participation [16, 17, 29, 34, 43, 45]. Three studies found that discourse and content analysis were applied to provide qualitative insights into communication patterns and the role of children in consultations [6, 8, 50]. One study by Aronsson and Rundström used child‐allocated turns (CATs), which refer to conversational turns that are explicitly directed or ‘allocated’ to the child [30]. Two studies used the Turn Allocation System (TAS) to identify the structured process by which conversational turns are distributed among participants during a discussion or interaction. It is crucial because it shapes how each participant is allowed to speak, respond, or contribute to the conversation [10, 38].
3. **Paediatric Dental Triadic Interaction Coding Scheme**
  In specific healthcare settings, customised coding schemes were developed to evaluate child participation. Only two papers in dental settings used the Paediatric Dental Triadic Interaction Coding Scheme to capture unique dynamics between children, parents, and dental providers; this coding scheme was applied to assess verbal and nonverbal child behaviours, such as affirmations, emotional responses, and prompt reactions [13, 44]. These tailored approaches offered context‐specific insights into child participation within specialised healthcare interactions.
4. **Other Instruments**
  Other studies have used different assessment methods to measure child participation in healthcare settings. A study by Bari et al. (2017) applied two specific assessment tools, which are the Paediatric Consultation Assessment Tool (PCAT) and the Arizona Clinical Medical Interview Rating Scale (ACIR) [32]. The PCAT is designed to evaluate how well healthcare services meet the needs of the patient, focusing on communication and shared decision‐making. ACIR measures the extent to which children are involved in the healthcare consultation, ensuring their voices are heard and considered. A study by Tates et al. used a 3‐point scale to measure child involvement in terms of their level of participation and engagement during medical consultation [49]. The scale categorises participation as active involvement, passive involvement or no display of involvement based on observable child behaviour. One study focused on measuring and capturing detailed verbal contributions of children during their healthcare interactions [41]. This method categorised the types of children's verbal responses, such as questions, statements about their feelings or descriptions of their symptoms, providing insight into their engagement level [41]. A study by Runeson et al. (2002) used a scale of participation in decision‐making as a tool to assess hospitalised children's participation in decision‐making and how they are involved in decisions about their treatment and care [48]. This approach allows for a deeper understanding of the communication and decision‐making dynamics at play in healthcare settings. A study by Forner et al. (2020) used multiple measures, behavioural, interactional coding, and questionnaire‐based assessment, to evaluate SDM‐related outcomes [26]. The study combined Roter Interaction Analysis System (RIAS) utterance‐level coding with turn‐taking/sequence analysis to examine how SDM and patient‐centred communication occurred during clinical interactions. These communication measures were complemented by a Patient‐Centeredness Score and an SDM questionnaire to capture patients' perceptions of the decision‐making process [26].

#### 3.2.3 Adults' Communication Strategies and Styles

The findings show that healthcare providers (HCPs) and caregivers use different communication strategies that influence child participation in healthcare consultations. Regarding HCPs' communication strategies, 18 papers reported a dyadic approach (instrumental, directive or task‐focused style between two parties) where HCPs mainly prioritised medical tasks, used medical terms, controlled the conversation and primarily addressed parents rather than children, limiting opportunities for children to engage [5, 6, 12, 26, 32, 34, 35, 36, 41, 42, 46, 48]. Dyadic approaches that control the dialogue and direct discussion toward parents instead of children undermine opportunities for children's active participation and tend to limit children's verbal participation and engagement [8, 16, 17, 28, 30, 38].

Conversely, 16 papers highlighted that HCPs who employed a triadic approach (facilitative and supportive style with a focus on rapport‐building) created a supportive environment that encouraged child involvement [43]. Healthcare providers who used supportive methods that actively engaged children by directly addressing them and encouraging their responses [10, 11, 31, 40]. For example, studies found that when HCPs used direct questions and gave children more space to respond, children participated more [27, 29, 33, 39, 45]. In dental settings, Yuan and colleagues showed that using age‐appropriate language at the child's developmental level and affirming their contributions through praise helped children understand, feel more comfortable, and be willing to participate [13, 44] By adopting a more facilitative approach, an environment that empowers children to participate actively in discussions about their health, which may positively influence their overall treatment experience [23, 25]. For instance, open and empathetic communication during oncological consultation is associated with a more positive experience for the child, while a dyadic directive, task‐oriented approach can contribute to feelings of anxiety and alienation [4, 24]. Overall, these findings suggest that supportive rapport‐building approaches can improve engagement, while dyadic or task‐focused communication styles may restrict children's participation.

For caregivers' communication strategies, 12 papers indicated that parents often act as supporters or advocates, helping children participate by explaining information and encouraging their input [10, 13, 24, 28, 34, 36, 38, 40, 41]. For example, studies found that children were more likely to join in conversations when parents validated their responses and clarified information [33, 43]. Some parents facilitate their children by rephrasing questions and providing emotional support, which allows them to feel more at ease and willing to speak up during consultations [44]. However, 14 papers showed that some parents take a more dominant role, controlling conversations and often speaking on behalf of the child, which can limit children's involvement [6, 8, 11, 12, 16, 17, 23, 26, 31, 32, 42, 45, 46, 48]. A protective, gatekeeping role was observed in three papers where parents controlled what information was shared with their child to shield them from distressing details [29, 35, 47]. Other papers highlight that parents exhibit varying approaches in facilitating or influencing their children's participation in healthcare settings [4, 5, 17, 25, 27, 30, 39, 50].

#### 3.2.4 Contextual Factors Influencing Child Participation

As mentioned above, child‐related factors (i.e. age and development) and HCPs' and caregivers' communication strategies have been shown to significantly impact child participation during the triadic consultation. In addition, we have found three other key factors that influence children's participation in healthcare consultations.

1. **Consultation context**
  The consultation context, including the type and complexity of the medical consultation, the clinic environment and visit length, influences child engagement within triadic interactions. Twelve studies highlighted that children were generally more active participants in routine consultations, which often involved casual or less technical discussions, such as social talk about school, hobbies, or daily activities [6, 8, 12, 13, 15, 26, 31, 32, 37, 40, 48]. However, Runeson et al. (2002) found that in complex consultations where detailed medical information needs to be conveyed, parents and healthcare providers tend to dominate the conversation, leaving less room for the child's involvement [48]. This highlights how the nature of the consultation can significantly influence the level of a child's engagement. Additionally, Forner et al. (2020) found that primary care consultations, especially initial ones, are generally shorter and devote less time to discussing broader health topics such as lifestyle compared to surgical specialities [26]. In other words, time constraints in primary care may limit deeper conversations about overall well‐being, which could reduce opportunities for children and families to engage more fully in their health discussions.
  Consultation environment and structure have been identified as a factor in 11 studies [4, 9, 13, 24, 28, 34, 36, 38, 44, 50, 53]., where structured environments that include the child, such as direct seating arrangements, were found to support greater participation, as reported by Meeuwesen and Kaptein, Cahill and Papageorgiou and Jenkins et al. [34, 36, 38]. Moreover, a welcoming and child‐friendly environment can significantly enhance a child's comfort level during a medical visit. When children feel safe and at ease, they are more likely to engage in conversations to ask questions and express their thoughts or concerns [53]. A supportive atmosphere can encourage children to participate more actively rather than remaining passive or hesitant [44]. However, in high‐stress or less supportive situations, as observed by Baggens and Coyne et al., children tend to participate less actively [4, 28].
  Four studies found visit length as another factor influencing child participation in healthcare consultations [14, 31, 37, 40]. Longer consultations provide healthcare providers with more time to interact with the child. This additional time allows for more in‐depth discussions, where children can be invited to share their feelings, ask questions, and contribute to the conversation. For example, Sleath et al. (2011) found that children were more likely to ask questions about asthma medications during longer visits [40]. This indicates that extra time not only facilitates rapport building but also encourages active participation. In contrast, shorter visits may rush the interaction, leaving little room for children to engage meaningfully.
2. **Cultural background**
  Findings from the three included studies revealed that cultural factors influence child participation within healthcare consultations [4, 12, 17]. Vigilante et al. (2015) found that cultural factors, along with the child's age and race/ethnicity, influenced child engagement during healthcare visits [12]. It was noted that children from different cultural backgrounds might have varying levels of comfort in terms of participating in the consultations. For instance, in many Asian cultures, children are often expected to defer to their parents or healthcare providers, which can limit their willingness to speak up during consultations. This contrasts with Western cultures, where children are encouraged to express their thoughts and actively participate in discussions about their care [17]. Different cultures have varying expectations regarding children's role in healthcare discussions. Furthermore, the role of parents in facilitating or hindering child participation is also influenced by cultural norms. For example, a study by Coyne et al. (2016) highlighted that in some cultures, parents may adopt a protective, gatekeeping role, controlling what information is shared with their child to shield them from distressing information [5]. Conversely, in cultures that promote open communication, parents may encourage their children to ask questions and express their feelings, leading to active child engagement during their healthcare consultation.
3. **Child psychological and emotional factors**
  Apart from children's age and development mentioned previously, psychological and emotional states have been shown to have an impact on children's participation in healthcare consultations. Psychological barriers such as anxiety and fear can significantly limit children's willingness to participate in consultations and actively discuss their health [34, 47]. Young children often exhibit reduced engagement in high‐stress situations, such as during invasive procedures, where fear can lead to withdrawal from conversations [4, 44]. When children felt safe and secure, their participation and engagement increased [24, 33]. Children who reported feeling anxious were less likely to ask questions or express their concerns, highlighting the need for healthcare providers to recognise and address these emotional states [25, 31].

#### 3.2.5 Lack of Studies in Dental Settings

Notably, although dentistry is a clinical setting in which children regularly have invasive treatments such as injections, extractions, and restorative work and often find themselves in very anxious environments, only a small number of the studies included were carried out in paediatric dental settings (4 out of 41) [13, 27, 33, 44]. Arguably, these features make communication and participatory practice at least as consequential in dental care as in general paediatric healthcare, yet the comparative scarcity of dental‐setting research identified in this review suggests that communication and participation practices in paediatric dentistry remain comparatively under‐examined. It points to an evidence gap that warrants dedicated empirical attention.

## 4 Discussion

Previous research by Tates and Meeuwesen (2001) reported child verbal contribution rates between 2% and 14% [54]. Similarly, a recent scoping review by Van Woerden et al. (2023) found that child talk ranged from 4% to 14% [55]. Our findings indicate that children's UNCRC participation rights are not consistently upheld in practice across the settings examined. Although professionals broadly endorse participatory communication in principle, the persistent gap between rhetoric and reality means that many child patients are still not meaningfully involved in decisions about their own care. This finding is consistent with the broader literature; Moore and Kirk (2010) similarly found that while children's participation rights were recognised in policy, they were inconsistently upheld in clinical practice, with protection concerns often overriding participation without sufficient guidance for practitioners [56]. More recently, Koller et al. (2024) extended this critique through a critical review of paediatric healthcare teams. They found that although there is growing recognition of children's voices, empowerment, access to information, and involvement in shared decision‐making, these practices are still rarely framed explicitly as children's rights under the UNCRC [51]. Taken together with our findings, these converging reviews underscore that the failure to uphold participation rights is a sustained, systemic issue across healthcare settings. This raises important questions about whether current communication practices truly support meaningful child involvement. Therefore, this review aimed to explore how children participate in triadic healthcare encounters involving doctors and caregivers. It examined the measures used to assess child participation and investigated how adults' communication styles, strategies, and contextual factors influence the level and quality of children's involvement.

### 4.1 Children's Participation in Triadic Consultation

The majority of studies (37 out of 41) indicate that child participation in triadic healthcare consultations is often limited. Children tend to have low verbal contributions or play passive roles during these interactions. For instance, Cox et al. (2009) found that children's talk accounted for only 6.7% of the total conversation [11]. A study by Vigilante et al. (2015) noted that children mostly responded briefly with simple words like ‘yes’ or ‘no’, or minimal explanations when prompted [12]. Similarly, Becker et al. (2018) observed that children rarely initiate conversations or ask questions [23]. This limited participation may result from several factors. Older children are more likely to engage actively, asking questions and contributing, whereas younger children tend to be more passive and reliant on caregivers to communicate on their behalf. The limited child participation appears to stem not only from the child's developmental stage but also from other systemic factors. In triadic consultations, the communication dynamic is often guided or dominated by healthcare providers. Thus, caregivers may struggle to navigate the balance between supporting their child's involvement and deferring to the healthcare professional (HCP).

### 4.2 Measures Used to Assess Child Participation

Measures used to assess child participation in healthcare consultations vary across studies and include both quantitative and qualitative approaches. Eight studies found that the Roter Interaction Analysis System (RIAS) is a widely used quantitative tool for measuring child participation in healthcare consultations. The assessment of child participation in healthcare consultations reveals a significant gap in quantitative measurement tools, as noted in the study by Cox et al. (2009) [11]. While Cox et al.'s finding that children only contributed 6.7% of visit talk provides a clear, quantifiable insight into children's engagement, later studies have primarily relied on qualitative or observational methods. This reliance on qualitative approaches allows for rich, nuanced understandings of the factors that influence child participation, including communication styles, cultural factors, and the consultation context. However, the lack of robust and standardised, widely used quantitative tools limits the ability to quantify and compare levels of child participation across different studies, settings, or over time. The heterogeneity in measurement tools hampers the development of evidence‐based strategies to enhance child involvement within triadic consultation, as it remains unclear how specific interventions influence participation levels over time. Overall, advancing child participation research would benefit from developing and consistently applying mixed methods approaches that combine the richness of qualitative data with the rigour and comparability of quantitative metrics.

### 4.3 HCPs' Communication Styles and Strategies During Triadic Consultation

The review highlights the significant impact of healthcare providers' communication styles and strategies on child participation during consultations. Eighteen studies reported that directive, task‐focused communication styles by healthcare providers, often using medical jargon or dyadic instructions (doctor–parent), tended to suppress child participation. When healthcare providers use complex medical terminology, children may find it difficult to understand the conversation, leading to feelings of confusion or discouragement from asking questions because they feel they are not equipped to contribute meaningfully. A study by Coyne et al. (2006) observed that medical jargon creates a barrier to effective communication, especially for children with limited health literacy. This can reflect discomfort or an inability to participate [5, 6, 30]. Many healthcare providers rely on dyadic interactions with parents, which often dominate the consultation and limit opportunities for children to ask questions, express their views or participate in shared decision‐making [28, 35, 38, 42]. This often places children in a passive role, where they are expected to respond to prompts without initiating dialogue themselves. For instance, a study by Bari et al. (2017) demonstrates that when the doctor takes a leading role in the interaction, they treat the patient as a medical object [41]. This dynamic can reflect a power imbalance, where the doctor is in control, and the child's participation is ignored, often disregarding non‐verbal cues or attempts to engage.

In contrast, sixteen studies found that facilitative, supportive approaches enhanced children's involvement in discussions about their care. Studies have observed that healthcare providers who adopt a supportive approach, using facilitative communication strategies characterised by directly addressing children by name and making explicit efforts to include them in the discussion, rather than speaking to caregivers, are more effective [10, 25, 31, 33, 39, 40, 44]. A study by Bhatti et al. (2021) explored that using age‐appropriate language that incorporates visual tools or playful techniques tailored to the child's development level helped children better understand and feel comfortable participating. A study by Yuan et al. (2019*)* found that the application of facilitative communication practices, such as dentally engaging talk, praise and ‘Tell‐Show‐Do’ instructions, promotes children's understanding, reduces anxiety and active participation [13, 44]. Studies indicate that Interactive strategies, such as open‐ended ones, are more effective than closed‐ended inquiries [33, 38, 40, 41]. When Healthcare Providers ask questions like ‘How are you feeling today?’, they invite children to express themselves freely rather than restrict them to simple ‘yes’ or ‘no’ answers [6, 31, 34, 39, 45]. It helps HCPs gain more comprehensive insights into children's experiences, emotions and concerns [6, 33, 39]. Moreover, it has been found that employing tag questions directed to children during paediatric palliative care consultations engages the children and improves their agency (e.g., ‘You're comfortable with that, aren't you?’). These questions encourage children to participate actively by inviting them to confirm or refuse without burdening them if they are not willing to respond [29]. It has been revealed that incorporating two or more effective communication strategies to address the child's specific needs would improve child participation. These strategies further support children's understanding and help them feel valued and involved; therapy reduces anxiety and empowers them to take a more active role in their healthcare [6, 45].

Parents play an important role in shaping their child's participation in healthcare settings, often acting as the ‘go‐between’ between the child and healthcare providers. Supportive parents can encourage their children to express their thoughts and feelings. Studies showed that a more supportive style of parenting could allow children to express themselves more freely [14, 17, 24]. While those who dominate the conversation can inadvertently stifle their child's voice [8, 10, 48, 57]. These findings suggest that parents' roles vary from supportive facilitators to protective gatekeepers, affecting how actively children participate in healthcare discussions. These dynamics raise important questions about the need for healthcare providers to engage not only with children but also with their parents, fostering a treatment alliance that both parties feel empowered to contribute to the discussion. [14].

Cultural contexts highlight the significant impact of cultural beliefs and practices on child engagement in healthcare. Different cultures have varying expectations regarding children's roles in healthcare discussions, which can influence how children perceive their ability to participate [4]. For instance, in some cultures, children are expected to withdraw from discussions, while in others, they are encouraged to take an active role in their care. This cultural variability necessitates a nuanced understanding of the diverse backgrounds of patients and their families, prompting healthcare providers to adopt culturally sensitive communication strategies that accommodate these differences. By addressing cultural factors, healthcare professionals can create a more inclusive environment that fosters child participation. Moreover, emotional support has been found to enhance child participation [24, 25, 34, 44, 47]. Research found that healthcare providers' supportive and empathetic approach could significantly reduce children's anxiety, leading to increased verbal participation during consultation [24, 44]. It is therefore important to create a welcoming and supportive environment to alleviate children's fear and anxiety [33].

Our review identified various tools to assess child participation in the triadic consultations [15]. Despite the frequent use of RIAS as a potentially standardised approach to measuring child participation, its broad, structured coding framework may miss the subtle, unique interactions found in paediatric settings [11, 12, 15, 23, 26, 31, 46, 47]. Customised measures for specific settings, such as PaeD‐TrICS, have the value of capturing detailed communication behaviours that are otherwise overlooked in more general assessments [44]. Nevertheless, the lack of standardised tools makes it challenging to compare findings and to assess the effectiveness of interventions to improve child engagement [23]. By establishing validated assessment tools, researchers and practitioners can better understand the factors that influence child participation and develop targeted strategies to enhance engagement in healthcare settings [31].

### 4.4 Research Implications

Healthcare professionals should adopt an inclusive approach that actively encourages children to voice their opinions and ask questions. Training programmes should be developed to equip healthcare providers with the skills necessary to communicate effectively with children, including the use of child‐friendly language and techniques that foster a supportive environment. Additionally, practitioners should engage parents in discussions about the importance of child participation, helping them understand how to facilitate their child's involvement rather than dominating the conversation. Further empirical research should investigate the interaction dynamics between children, parents, and healthcare providers during consultations, exploring the specific barriers and facilitators that influence child engagement and communication. Moreover, using validated tools could help track progress and identify areas for improvement. These validated metrics accurately measure the impact of child participation on healthcare delivery and outcomes, allowing for comparative studies across different settings and populations.

### 4.5 Limitations

This scoping review had a few limitations. First, it exclusively included papers published in English, which may have led to the omission of relevant studies from non‐English‐speaking countries. Second, in contrast to systematic reviews, scoping reviews do not evaluate the quality of each included study. Nonetheless, the application of the PRISMA‐ScR guidelines helped to mitigate this limitation to some extent. Lastly, while this review incorporated only primary studies, care was taken to ensure that there was no double‐counting of findings, thus maintaining the integrity of the evidence presented.

## 5 Conclusion

This review highlights the critical need for enhanced child participation in healthcare consultations, which emphasises the importance of effective communication strategies that empower children to engage actively in their care. However, addressing this gap will take more than just communication skills training. Health professionals need to work closely alongside parents or caregivers as advocates for children's participation rights. Without parental support, health professionals will struggle to translate these participatory principles into their daily clinical practice. Thus, it is essential to engage both professionals and parents together to drive clinical practice changes through a fundamental cultural shift in terms of paediatric care delivery. Future research should employ validated measurement tools to evaluate progress and identify effective approaches for enhancing child engagement in healthcare settings. Prioritising both research and practice that foster inclusive environments where children's voices are heard, respected, and valued will ultimately contribute to improved healthcare experiences and better health outcomes for young patients.

## Author Contributions

**Maha Alharbi:** conceptualisation, methodology, software, formal analysis, writing – original draft, data curation. **Suzanne Grant:** conceptualisation, supervision, writing – review and editing, methodology, validation. **George Cherukara:** supervision, conceptualisation, methodology, writing – review and editing, validation. **Siyang Yuan:** writing – review and editing, supervision, methodology, conceptualisation, validation.

## Ethics Statement

No ethical approval is needed, given the nature of a scoping review. The data are retrieved and analysed based on the previously published studies in which informed consent was obtained by the primary investigators.

## Conflicts of Interest

The authors declare no conflicts of interest.

## Supporting information


Supporting File

## Data Availability

The data that support the findings of this study are available from the corresponding author upon reasonable request.
