# Supplementary material for: Communication Strategies to Improve Child Participation in Triadic Consultations Within Healthcare Settings: A Scoping Review
Source: Health Expect. 2026 Aug 26;29(5):e70853. doi: 10.1111/hex.70853 (PMC13518228; doi:10.1111/hex.70853)
Supplement: Supplementary file 1 [file HEX-29-e70853-s001.docx]

**Appendix**

**Search Strategies including keywords or Medical Subject Heading (MeSH) terms**

| **Medline via PubMed** | **APA PsycINFO** | **CINAHL** | **Scoups** |
| --- | --- | --- | --- |
| (((Child[Mesh] OR Adolescent[Mesh] OR Child*[Title] OR Adolescen*[Title] OR Paediatric[Title] OR Pediatric[Title]) AND (Patient participation[Mesh] OR Physician-Patient Relations[Mesh] OR Decision making[Mesh] OR Shared-decision making[Title/Abstract] OR Decision making[Title/Abstract] OR Patient-centred[Title/Abstract] OR Patient-centered[Title/Abstract] OR Person-centred[Title/Abstract] OR Person-centered[Title/Abstract] OR Patient-focused[Title/Abstract] OR Participation[Title/Abstract] Involve*[Title/Abstract] OR Engagement[Title/Abstract] OR Interaction*[Title/Abstract])) AND (Referral and Consultation[Mesh] OR Consultation*[Title/Abstract] OR Encounter*[Title/Abstract])) AND (Communication[Mesh] OR Communicat*[Title/Abstract] OR Talk*[Title/Abstract] OR Verbal[Title/Abstract] OR Non-verbal[Title/Abstract] OR Listen*[Title/Abstract] OR Speak*[Title/Abstract] OR Discourse[Title/Abstract] OR Conversation*[Title/Abstract] OR Language[Title/Abstract]) | 1.(Child* or Adolescen* or Paediatric or Pediatric).ti,ab.  2.("Patient participation" or "Decision making" or "Shared-decision making" or Patient-centred or Patient-centered or Person-centred or Person-centered or Patient-focused or Participation or Involve* or Engagement or Interaction*).ti,ab.  3.(Consultation* or encounter*).ti,ab.  4.(Communicat* or Talk* or Verbal or Non-verbal or Listen* or Speak* or Discourse or Conversation* or Language).ti,ab.  5.(nurse* or doctor* or "healthcare professional*" or "health care professional" or physician or "healthcare practitioner*" or "health care practitioner" or dentist* or clinician or GP or "General practitioner" or Consultant* or Pediatrician or Paediatrician).ti,ab.  6.1 and 2 and 3 and 4 and 5 | AB ( Child* OR Adolescen* OR Paediatric OR Pediatric ) AND AB ( “Patient participation” OR “Decision making” OR “Shared-decision making” OR Patient-centred OR Patient-centered OR Person-centred OR Person-centered OR Patient-focused OR Participation OR Involv* OR Engagement OR Interaction* ) AND AB ( Consultation* OR Encounter* ) AND AB ( Communicat* OR Talk* OR Verbal OR Non-verbal OR Listen* OR Speak* OR Discourse OR Conversation* OR Language ) | ( TITLE ( child* OR adolescen* OR paediatric OR pediatric ) AND TITLE-ABS-KEY ( "patient participation" OR "decision making" OR "shared-decision making" OR patient-centred OR patient-centered OR person-centred OR person-centered OR patient-focused OR participation OR involv* OR engagement OR interaction* ) AND TITLE-ABS-KEY ( consultation* OR encounter* ) AND TITLE-ABS-KEY ( communicat* OR talk* OR verbal OR non-verbal OR listen* OR speak* OR discourse OR conversation* OR language ) AND TITLE-ABS-KEY ( nurse* OR doctor* OR "healthcare professional*" OR "health care professional" OR physician OR "healthcare practitioner*" OR "health care practitioner" OR dentist* OR clinician OR gp OR "general practitioner" OR consultant* OR pediatrician OR paediatrician ) ) |
